# Cutaneous botryomycosis in sequelae lesions of cutaneous leishmaniasis: case report in Peru

**DOI:** 10.17843/rpmesp.2026.432.15488

**Published:** 2026-06-10

**Authors:** Jossy Elena García-Ubillús, Josué M. Flores-Espejo, Rafael Alarco Herrera, Roger Sernaque Mechato

**Affiliations:** 1 Facultad de Medicina Humana, Universidad de Piura, Lima, Peru.; 2 Departamento de Medicina Interna, Hospital Santa Rosa, Lima, Peru.

**Keywords:** Staphylococcus haemolyticus, Botryomycosis, Mycetoma, Skin Diseases, Bacterial, Actinomycosis

## Abstract

Botryomycosis is a rare chronic bacterial infection characterized by bacterial granules in an eosinophilic matrix. We present the case of a 47-year-old male with progressive cutaneous lesions, initially treated as leishmaniasis without favorable clinical response. The diagnosis of cutaneous botryomycosis was confirmed by the identification of the Splendore-Hoeppli phenomenon in the biopsy. Additionally, the isolation of *Staphylococcus haemolyticus* guided the antibiotic treatment, achieving a partial response. This case highlights the importance of considering botryomycosis within the differential diagnosis of chronic cutaneous lesions, especially in endemic areas for infections with similar clinical presentations.

## INTRODUCTION

Botryomycosis is a chronic, granulomatous, and suppurative bacterial disease that affects the skin and viscera. The most common etiological agent is *Staphylococcus aureus*, followed by *Pseudomonas aeruginosa*, *Escherichia coli*, and other microorganisms ^1^. The most frequent clinical presentation is cutaneous, characterized by solitary or multiple suppurative nodular lesions that usually affect exposed areas, especially the lower limbs ^2^.

It is a rare disease, with approximately 200 cases reported until 2012, mainly in the United States and Europe ^3^. In Peru, the literature describes two cases in the northern zone ^4^, one in Ayacucho in a patient coinfected with HTLV-1 and *Strongyloides stercoralis*^5^ and another in Junín, in an adolescent with a four-year disease duration ^6^. The reduced number of reported cases could be explained because it is an infection frequently underdiagnosed and confused with other chronic pathologies, such as mycetomas, neoplasms, and tuberculosis.

This report presents the case of an immunocompetent patient, with a previous leishmaniasis infection, in whom cutaneous botryomycosis was diagnosed after ruling out mycotic infections and after being confused with leishmaniasis. In this case, the histopathological diagnosis allowed for timely treatment and prevented extension to bone tissue, highlighting the diagnostic challenges in a disease infrequent in our environment.

## CASE REPORT

### Patient information

A 47-year-old male patient, mixed-race, from the district of Rioja (department of San Martin), engaged in fishing and agriculture, with no significant medical history. He was admitted to Santa Rosa Hospital via the infectology outpatient clinic in March 2025. He presented with a three-year history of illness, with subacute onset and progressive course, which began with a single 1 × 2 cm pustule located in the lateral region of the left knee. The lesion was treated with the application of ointments for five months, without evidencing clinical improvement.

On September 5, 2024, he attended a health center in Rioja due to the progression of the lesion, which had ulcerated and presented violaceous borders, three central scabs, and regional throbbing pain. A tissue sample was obtained by scraping and a direct microscopic examination was performed, which tested positive for cutaneous leishmaniasis. He was treated with three daily ampules of N-methyl glucamine antimoniate (1215 mg) for 20 days. On October 18, he was reevaluated, showing no clinical improvement. Therefore, a new course of N-methyl glucamine antimoniate was administered to him, increasing the dose to four daily ampules (1620 mg) for 20 days.

Despite completing the second course of treatment, the lesions spread to the entire popliteal region and the external aspect of the lower third of the left thigh. Clinically, they progressed to nodules with ulcerated centers and superficial abscesses, associated with paresthesias and functional limitation. Due to this unfavorable evolution, on January 25, 2025, the patient returned for a new smear, whose result was negative for active cutaneous leishmaniasis. Subsequently, on January 30, a serological test was performed by indirect immunofluorescence, which did not detect anti-*Leishmania* IgG antibodies.

Due to the unfavorable evolution of the lesions, the patient attended on March 25 the infectious diseases outpatient clinic of Hospital Santa Rosa, in Lima, Peru. He had a previous wound culture that identified *Staphylococcus haemolyticus* susceptible to chloramphenicol, rifampicin, teicoplanin, and linezolid.

### Clinical findings

On physical examination, nodular plaques and irregular-edged ulcers approximately 10 × 8 cm in size, some with serohematic crusts and purulent discharge, were observed on the medial aspect of the distal third of the left thigh. The lesions presented with erythematous and indurated borders, in addition to satellite pustular lesions extending towards the popliteal region. Upon digital pressure, the lesions were painful and suppurative (figure 1). Additionally, the patient reported stabbing pain and limitation for flexion of the left limb.

**Figure 1:**
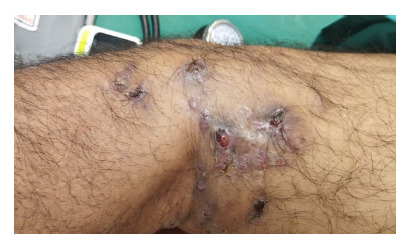
Multiple ulcerated lesions on the posterior aspect of the left knee, with violaceous borders and areas of seropurulent discharge compatible with cutaneous botryomycosis.

### Diagnostic approach

On hospitalization day (HD) 1, probably abscessed myositis was suspected, so an interconsultation to the traumatology service was requested for surgical debridement and culture collection. Laboratory tests (blood count, glucose, urea, and creatinine) were within reference ranges. Serology for VDRL, surface antigen, and HIV were non-reactive.

### Differential diagnoses

Among the main differential diagnoses, leishmaniasis was considered due to the presence of multiple nodular and ulcerated lesions, as well as a possible history of recurrent infection. Cutaneous tuberculosis, nocardiosis, and actinomycosis were also evaluated due to their association with fistulous tracts and purulent discharge.

Another differential diagnosis was sporotrichosis, which can manifest with chronic nodules, infiltrated lesions, and lymphatic tracts, especially given the possibility of trauma with thorns due to his farming history. Finally, although less probable, a soft tissue sarcoma was considered, as some can ulcerate and necrose; however, computed tomography and magnetic resonance showed only subcutaneous edema, inflammatory epidermal thickening, compartmental collections, and myositis, without solid masses or bone damage, ruling out a malignant etiology. The skin biopsy reported exacerbated chronic inflammatory infiltrate and granulation tissue, without evidence of neoplasia.

### Evolution

On HD 3, a staphylococcal superinfection was considered due to the positivity of the previous culture. On HD 7, the patient entered the operating room for surgical debridement and sample collection. During the procedure, fibrous tissue approximately 10 × 8 cm was found, without purulent discharge. The obtained samples were sent for aerobic, anaerobic cultures, acid-fast bacilli, fungi (including sporotrichosis), and histopathology.

Cultures for anaerobes and BK were negative, with the main differential diagnoses remaining a mycosis (sporotrichosis or actinomycosis) and a staphylococcal superinfection. On HD 11, the fungal culture was also negative. Histopathological study showed fibroconnective tissue with dense neutrophilic infiltrate, granulation tissue, multinucleated giant cells, and bacterial granules with eosinophilic rim, compatible with Splendore-Hoeppli phenomenon (figure 2). With these findings, the diagnosis of cutaneous botryomycosis was confirmed.

**Figure 2:**
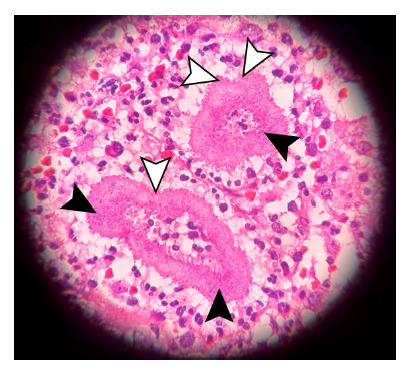
Microphotograph of connective tissue at 400X with hematoxylin-eosin staining. The Splendore-Hoeppli phenomenon is observed: basophilic granules (black arrowhead) surrounded by an eosinophilic matrix (white arrowheads). Courtesy of the Anatomic Pathology Service of Santa Rosa Hospital.

### Therapeutic approach

On DH1 the patient started treatment with ceftazidime 2 g IV every 8 hours, clindamycin 600 mg IV every 8 hours, and paracetamol 1 g PO as needed for pain for 5 days. This regimen was initially chosen because a *Pseudomonas* infection was considered (reason for ceftazidime use) and, due to the previous culture with *Staphylococcus haemolyticus*, clindamycin was added. However, on the fifth day without clinical improvement, methicillin-resistant *Staphylococcus aureus* infection was suspected. Therefore, clindamycin was discontinued and vancomycin was started (2 g IV loading dose, followed by 1 g every 12 hours) for three effective days, until surgical debridement, with ceftazidime maintained until completing nine effective days.

During the surgical intervention, debridement of fibrous tissue was performed, evidencing abundant serosanguineous gelatinous material and fibrosed muscle. Samples were taken for culture and histopathology, and subsequently, a profuse lavage with saline solution and adequate hemostasis were performed. Vacuum-assisted closure was not indicated because there was no significant tissue loss or muscle necrosis that warranted extensive debridement; furthermore, there was no active bleeding or exudate that justified negative pressure therapy.

On HD 11, after receiving the histopathological report and considering the sensitivity of the previously isolated *Staphylococcus*, linezolid 600 mg PO every 12 hours for 10 days (two of which were administered during hospitalization) was prescribed, associated with celecoxib 200 mg PO every 12 hours and daily wound care. Linezolid was chosen due to its excellent oral bioavailability, equivalent to the intravenous formulation, which allowed the treatment to be continued on an outpatient basis. In contrast, vancomycin requires close monitoring of renal function and plasma levels, while rifampicin demands monitoring of hepatic function, aspects that are hardly feasible in a patient from a rural area with difficulty attending frequent check-ups. The antimicrobial susceptibility profile of the isolate is presented in the supplementary material.

### Follow-up and Resultsfigure 4

The patient was discharged after 14 days of hospitalization. At a three-month post-intervention follow-up, a scar approximately 10 cm long was observed, with adequately approximated edges, in addition to persistence of two ulcers in the lesion and partial remission of induration (figure 3). The patient reported slight improvement in pain and persistence of functional limitation when walking.

**Figure 3:**
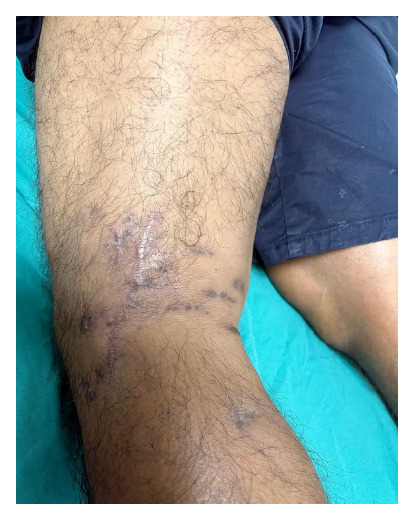
Three-month post-intervention follow-up. An irregular linear scar is observed on the right knee with residual hyperpigmented macules.

**Figure 4:**
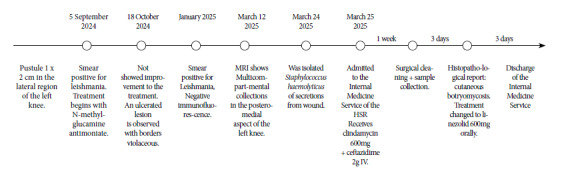
Timeline showing the patient’s disease history from onset to discharge from the service.

MRI: nuclear magnetic resonance, HSR: Santa Rosa Hospital

## DISCUSSION

Botryomycosis is part of the entities called pseudomycoses, a group of chronic infections that appear to be caused by fungi but have a bacterial origin. This disease usually occurs more frequently in immunosuppressed patients or with comorbidities, such as diabetes mellitus, chronic liver disease, prolonged corticosteroid use, malnutrition, autoimmune diseases, and acquired immunodeficiency syndrome ^3^^,^^7^.

The pathophysiology of botryomycosis is not yet fully understood; however, most reported cases occur after an inoculating trauma in exposed areas such as the head, neck, and extremities, which triggers an imbalance between the host's response factor to the foreign agent. An intermediate inoculum with low-virulence bacteria favors the chronification of the lesion and the formation of granules. These are microscopically characterized by the Splendore-Hoeppli phenomenon, an eosinophilic peripheral hyaline matrix (IgG and C3 complement deposits) that surrounds a basophilic center (non-filamentous bacteria) ^8^.

Two forms of the disease have been described: cutaneous and visceral. The cutaneous form usually manifests with solitary or multiple nodular lesions, abscesses, fistulas, or ulcers. They may present with purulent discharge, white-yellowish granules, or indurated plaques, which may extend to muscle or bone ^4^. The visceral form is less frequent, and its main location is pulmonary. The most common symptom is recurrent episodes of hemoptysis. Therefore, it is often initially confused with tuberculosis, actinomycosis, aspergillosis, or pulmonary neoplasms ^9^.

The diagnosis of botryomycosis is established by identifying non-filamentous bacteria from clinically compatible lesions. It can be performed through microscopy of the secreted granules, culture of the purulent exudate, or biopsy of the affected tissue ^10^. It is recommended to use potassium hydroxide (KOH), Giemsa, Ziehl-Neelsen stains, among others; as well as to culture in agars for bacteria and fungi. In this way, other differential diagnoses can be ruled out, such as actinomycosis, mycetomas, tuberculosis, and nocardiosis ^11^.

The disease predominates in males and in middle-aged individuals. Most reported cases correspond to patients from rural areas, especially farmers, with risk factors such as smoking. Likewise, several reports describe as common antecedents the presence of previous trauma and a disease duration longer than 30 days ^3^^,^^11^^-^^14^.

In this case, the patient had a history of cutaneous leishmaniasis, which is why we propose that the initial lesions could have favored secondary infection by *Staphylococcus*, acting as an entry point for this microorganism habitually present on the skin. After receiving treatment for leishmaniasis, the staphylococcal infection would have persisted and progressed to cutaneous botryomycosis. This would explain that, after completing antiparasitic treatment, the smear was negative for leishmaniasis despite the persistence of clinically suggestive cutaneous lesions, initially maintaining the erroneous differential diagnosis of active leishmaniasis.

Another possibility is the simultaneous coexistence of both microorganisms, a situation that has been previously reported in Peru, where coinfections by *Staphylococcus* with *Peptoestreptococcus*, *Actinobacillus* and even with parasitic diseases such as *Strongiloides*^5^ have been described. From an epidemiological point of view, although he was a male patient from a rural area, he lacked risk factors such as diabetes or any immunosuppression state; likewise, during the interrogation, he denied a traumatic history prior to the onset of the condition reported in the literature.

Our finding matches a case reported in Lambayeque, in a farmer with right foot involvement due to dorsal fistulas with foul-smelling secretion and white-yellowish granules, of five years' evolution. In this case, *Staphylococcus aureus* and *Actinobacillus* were isolated, with metatarsal bone involvement. The patient received rifampicin 600 mg and co-trimoxazole every eight hours for two months, with partial improvement. However, he refused surgical treatment and did not attend subsequent follow-ups, which highlights the limitation of follow-up in prolonged therapies, given the possibility of abandonment due to initial improvements ^4^.

Regarding antibiotic treatment, it will depend on the availability and sensitivity profile of the isolated agent (*Staphylococcus haemolyticus)*; in the present case, linezolid 600 mg in tablets every 12 hours for 10 days was chosen; however, in another case, due to its sensitivity profile, the use of oral cefadroxil and clavulanic acid (500 mg, twice a day) for 10 days along with rifampicin (600 mg, once a day) for two weeks was chosen ^15^. A case report in Taiwan of an elderly patient with greater comorbidities than those in the present report also opted for surgical debridement of the affected region; however, in antibiotic therapy, intravenous vancomycin for three weeks was chosen ^16^. Both cases had a favorable evolution. Antibiotic treatment options with reported efficacy include erythromycin 500 mg, dicloxacillin 100 mg, trimethoprim-sulfamethoxazole 800/160 mg, cephalosporins, and clindamycin, administered for several weeks and in combination with surgical management ^3^^,^^6^^,^^11^^,^^17^. Among invasive treatments, the use of vacuum-assisted closure (VAC) for the drainage of secretions after initial surgical excision with good results should also be considered ^18^.

### Implications for public health

This case demonstrates that botryomycosis can be confused with Peruvian endemic pathologies such as leishmaniasis, which generates diagnostic delays and inadequate treatments. The presentation of this pathology in an immunocompetent patient emphasizes the importance of considering it within the differential diagnostic approach for chronic cutaneous lesions. Furthermore, the identification and susceptibility profile of *Staphylococcus haemolyticus* highlights the need to strengthen microbiological surveillance in the country, especially given the increase in strains with complex resistance profiles. Because a large part of the affected population comes from rural areas with limited access to health services, strategies are required that improve the availability of microbiological studies and promote the use of effective and accessible oral therapeutic regimens.

A relevant limitation for the reporting of this case was the incomplete and disorganized availability of laboratory tests and images obtained in the patient's previous consultations, which made it difficult for the authors to accurately reconstruct the diagnostic sequence and disease progression. Likewise, follow-up after discharge was limited due to the patient's difficulties in attending the final check-ups by the infectious diseases department, which restricted the long-term evaluation of the therapeutic response.

In conclusion, this report evidences the need to consider botryomycosis within the differential diagnosis of unspecified chronic granulomatous pathologies, even in the absence of classic risk factors. Likewise, it highlights the importance of contextualizing the diagnostic approach according to the epidemiology of each region. In this case, the clinical characteristics of the lesion initially pointed towards an endemic parasitic disease in Peru, such as leishmaniasis, which delayed the initiation of appropriate treatment. Furthermore, due to changing resistance profiles, it is essential to have an antibiogram that confirms the susceptibility profile of *Staphylococcus haemolyticus*, since standard antibiotic therapy usually employed in dermal infections (beta-lactams) could be insufficient. Finally, although it should be reserved as a last therapeutic alternative, surgical management should not be ruled out in cases of extensive lesions with poor response to antibiotic therapy.
