# Supplementary material for: Cutaneous botryomycosis in sequelae lesions of cutaneous leishmaniasis: case report in Peru
Source: Rev Peru Med Exp Salud Publica. 2026 Jun 10;43(2):267–72. doi: 10.17843/rpmesp.2026.432.15488 (PMC13456929; doi:10.17843/rpmesp.2026.432.15488)
Supplement: Supplementary file 1 [file rpmesp-43-02-15488-s001-es.docx]

**Material suplementario**

**Tabla 1.** Antibiograma (cultivo de secreción positivo a *Staphylococcus haemolyticus*)

| Análisis | resultado |
| --- | --- |
| Clasificación de antibióticos | |
| Penicilinas |  |
| Penicilinas (naturales, aminopenicilinas) | resistente |
| Penicilinas resistentes a Blactamasas (oxa, cloxa, dicloxa, meticilinas) | resistente |
| Penicilinas + inhibidores de B lactamasas (amoxiclina/acido clavulanico, ampicilina/sulbactam ) | resistente |
| Cefalosporinas |  |
| Cefalosporinas 1era generación | resistente |
| Cefalosporinas 2da generación | resistente |
| Cefalosporinas 3era generación | resistente |
| Cefixima | resistente |
| Cefotaxima | resistente |
| Ceftriaxona | resistente |
| Cefalosporinas 4ta generación | resistente |
| Cefepime | resistente |
| Carbapenem | resistente |
| Aminoglucósidos |  |
| Gentamicina | resistente |
| Amikacina | resistente |
| Macrólidos |  |
| Eritromicina | resistente |
| Claritromicina | resistente |
| Roxitromicina | resistente |
| Azitromicina | resistente |
| Fluoroquinolonas |  |
| Ciprofloxacina | resistente |
| Levofloxacina | resistente |
| Moxifloxacina | resistente |
| Otros |  |
| Tetraciclina | resistente |
| Sulfametoxazol-Trimetoprim | resistente |
| Cloranfenicol | sensible |
| Clindamicina/Lincomicina | resistente |
| Rifampicina | sensible |
| Teicoplanina | sensible |
| Linezolid | sensible |
| Vancomicina | sensible |
