# Supplementary material for: Cutaneous botryomycosis in sequelae lesions of cutaneous leishmaniasis: case report in Peru
Source: Rev Peru Med Exp Salud Publica. 2026 Jun 10;43(2):267–72. doi: 10.17843/rpmesp.2026.432.15488 (PMC13456929; doi:10.17843/rpmesp.2026.432.15488)
Supplement: Translation

# Translation: Botriomicosis cutánea en lesiones secuelares de leishmaniasis cutánea: reporte de caso en Perú

**Authors:** Jossy Elena García-Ubillús, Josué M. Flores-Espejo, Rafael Alarco Herrera, Roger Sernaque Mechato

**Affiliations:** 1 Facultad de Medicina Humana, Universidad de Piura, Lima, Perú.; 2 Departamento de Medicina Interna, Hospital Santa Rosa, Lima, Perú.

**Keywords:** Staphylococcus haemolyticus, Botriomicosis, Micetoma, Enfermedades de la Piel, Enfermedades Cutáneas Bacterianas, Actinomicosis

## Abstract

La botriomicosis es una infección bacteriana crónica poco frecuente, caracterizada por gránulos bacterianos en una matriz eosinófila. Presentamos el caso de un varón de 47 años con lesiones cutáneas progresivas, inicialmente tratadas como leishmaniasis sin respuesta clínica favorable. El diagnóstico de botriomicosis cutánea se confirmó mediante la identificación del fenómeno de Splendore-Hoeppli en la biopsia. Asimismo, el aislamiento de *Staphylococcus haemolyticus* guió el tratamiento antibiótico, logrando una respuesta parcial. Este caso resalta la importancia de considerar la botriomicosis dentro del diagnóstico diferencial de las lesiones cutáneas crónicas, especialmente en zonas endémicas de infecciones con presentaciones clínicas similares.

## INTRODUCCIÓN

La botriomicosis es una enfermedad bacteriana, crónica, granulomatosa y supurativa, que compromete la piel y vísceras. El agente etiológico más común es *Staphylococcus aureus*, seguido de *Pseudomonas aeruginosa*, *Escherichia coli,* y otros microrganismos ^1^. La presentación clínica más frecuente es la cutánea, caracterizada por lesiones nodulares supurativas, solitarias o múltiples, que suelen afectar zonas expuestas, especialmente los miembros inferiores ^2^.

Se trata de una enfermedad poco frecuente, con aproximadamente 200 casos reportados hasta 2012, principalmente en Estados Unidos y Europa ^3^. En el Perú, la literatura describe dos casos en la zona norte ^4^, uno en Ayacucho en una paciente coinfectada con HTLV-1 y *Strongiloides stercolaris*^5^ y otro en Junín, en una adolescente con un tiempo de enfermedad de cuatro años ^6^. El reducido número de casos reportados podría explicarse porque se trata de una infección frecuentemente infradiagnosticada y confundida con otras patologías crónicas, como micetomas, neoplasias y tuberculosis.

En este reporte se presenta el caso de un paciente inmunocompetente, con infección previa de leishmaniasis, en quien se diagnosticó botriomicosis cutánea luego de descartar infecciones micóticas y luego de ser confundida con leishmaniasis. En este caso, el diagnóstico histopatológico permitió brindar tratamiento oportuno y evitar la extensión hacia tejido óseo, resaltando los desafíos diagnósticos en una enfermedad infrecuente en nuestro entorno. 

## REPORTE DE CASO

### Información del paciente

Paciente varón de 47 años, mestizo, procedente del distrito de Rioja (departamento de San Martín), dedicado a la pesca y agricultura, sin antecedentes médicos de importancia. Ingresó al Hospital Santa Rosa por consultorio de infectología en marzo de 2025. Presentaba un tiempo de enfermedad de tres años, de inicio subagudo y curso progresivo, que comenzó con una pústula única de 1 × 2 cm localizada en la región lateral de la rodilla izquierda. La lesión fue tratada con la aplicación de pomadas durante cinco meses, sin evidenciar mejoría clínica.

El 5 de septiembre de 2024 acudió a un centro de salud en Rioja debido a la progresión de la lesión, la cual se había ulcerado y presentaba bordes violáceos, tres costras centrales y dolor punzante regional. Se obtuvo una muestra de tejido mediante raspado y se realizó un examen microscópico directo, que resultó positivo para leishmaniasis cutánea. Fue tratado con tres ampollas diarias de antimoniato de N-metil glucamina (1215 mg) por 20 días. El 18 de octubre fue reevaluado, evidenciándose ausencia de mejoría clínica. Por ello, se le administró un nuevo curso de antimoniato de N-metil glucamina, incrementando la dosis a cuatro ampollas diarias (1620 mg) por 20 días.

A pesar de completar el segundo ciclo de tratamiento, las lesiones se extendieron a toda la región poplítea y cara externa del tercio inferior del muslo izquierdo. Clínicamente, progresaron a nódulos con centro ulcerado y abscesos superficiales, asociados a parestesias y limitación funcional. Debido a esta evolución desfavorable, el 25 de enero de 2025, acudió nuevamente para la realización de un nuevo frotis, cuyo resultado fue negativo para leishmaniasis cutánea activa. Posteriormente, el 30 de enero se realizó una prueba serológica mediante inmunofluorescencia indirecta, la cual no detectó anticuerpos IgG anti-*Leishmania*.

Debido a la evolución desfavorable de las lesiones, el paciente acudió el 25 de marzo a la consulta externa de infectología del Hospital Santa Rosa, en Lima, Perú. Contaba con un cultivo previo de la herida que identificó *Staphylococcus haemolyticus* sensible a cloranfenicol, rifampicina, teicoplanina y linezolid.

### Hallazgos clínicos

En el examen físico se observaron, en la cara medial del tercio distal del muslo izquierdo, placas nodulares y úlceras de bordes irregulares de aproximadamente 10 × 8 cm, algunas con costras serohemáticas y con secreción purulenta. Las lesiones presentaban bordes eritematosos e indurados, además de lesiones pustulosas satélites que se extendían hacia la región poplítea. A la digitopresión, las lesiones eran dolorosas y supurativas (figura 1). Asimismo, el paciente refería dolor punzante y limitación para la flexión del miembro izquierdo.

**Figura 1:** Lesiones ulceradas múltiples en cara posterior de rodilla izquierda, con bordes violáceos y areas de secreción seropurulenta com-patibles con botriomicosis cutánea.

### Abordaje diagnóstico

En su día de hospitalización (DH) 1 se sospechó de miositis probablemente abscedada, por lo que se solicitó interconsulta al servicio de traumatología para limpieza quirúrgica y toma de cultivos. Los exámenes de laboratorio (hemograma, glucosa, urea y creatinina) se encontraron dentro de los rangos de referencia. La serología para VDRL, antígeno de superficie y VIH resultaron no reactivas.

### Diagnósticos diferenciales

Entre los principales diagnósticos diferenciales se consideró leishmaniasis, debido a presencia de múltiples lesiones nodulares y ulceradas, además de un posible antecedente de infección recidivante. También se evaluaron tuberculosis cutánea, nocardiosis y actinomicosis, por su asociación con trayectos fistulosos y secreción purulenta.

Otro diagnostico diferencial fue esporotricosis, que puede manifestarse con nódulos crónicos, lesiones infiltradas y trayectos linfáticos, especialmente ante la posibilidad de un traumatismo con espinas por su antecedente de agricultor. Finalmente, aunque menos probable, se planteó un sarcoma de partes blandas, ya que algunos pueden ulcerarse y necrosarse; sin embargo, la tomografía y la resonancia mostraron únicamente edema subcutáneo, engrosamiento epidérmico inflamatorio, colecciones compartimentales y miositis, sin masas sólidas ni daño óseo, descartando una etiología maligna. La biopsia de piel reportó infiltrado inflamatorio crónico reagudizado y tejido de granulación, sin evidencia de neoplasia.

### Evolución

En el DH 3 se consideró una sobreinfección por estafilococo, debido a la positividad del cultivo previo. En el DH 7, el paciente ingresó a sala de operaciones para limpieza quirúrgica y toma de muestras. Durante el procedimiento se encontró tejido fibroso de aproximadamente 10 × 8 cm, sin secreción purulenta. Las muestras obtenidas fueron enviadas para cultivos aerobios, anaerobios, bacilos ácido-alcohol resistentes, hongos (incluida esporotricosis) e histopatología.

Los cultivos para anaerobios y BK resultaron negativos, manteniéndose como principales diagnósticos diferenciales una micosis (esporotricosis o actinomicosis) y una sobreinfección por estafilococo. En el DH 11, el cultivo de hongos también fue negativo. En estudio histopatológico mostró tejido fibroconectivo con denso infiltrado neutrofílico, tejido de granulación, células gigantes multinucleadas y gránulos bacterianos con reborde eosinofílico, compatibles con fenómeno de Splendore-Hoeppli (figura 2). Con estos hallazgos, se confirmó el diagnóstico de botriomicosis cutánea.

**Figura 2:** Microfotografía de tejido conectivo a 400X con tinción de hematoxilina-eosina. Se observa el fenómeno de Splendore-Hoeppli: gránulos basófilos (cabeza de flecha negra) rodeados por una matriz eosinofílica (cabezas de flecha blancas). Cortesía del Servicio de Anatomía Patológica del Hospital Santa Rosa.

### Abordaje terapéutico

El DH1 el paciente inició tratamiento con ceftazidima 2 g EV cada 8 horas, clindamicina 600 mg EV cada 8 horas y paracetamol 1 g VO condicional a dolor por 5 días. Este esquema se eligió inicialmente porque se consideró una infección por *Pseudomonas* (motivo del uso de ceftazidima) y, debido al cultivo previo con *Staphylococcus haemolyticus*, se añadió clindamicina. Sin embargo, al quinto día sin mejoría clínica, se sospechó infección por *Staphylococcus aureus* resistente a meticilina. Por ello, se suspendió la clindamicina e inició vancomicina (2 g EV dosis de carga, seguida de 1 g cada 12 horas) durante tres días efectivos, hasta el lavado quirúrgico, manteniéndose la ceftazidima hasta completar nueve días efectivos.

Durante la intervención quirúrgica se realizó debridamiento de tejido fibroso, evidenciandose abundante material gelatinoso serosanguinolento y músculo fibrosado. Se tomaron muestras para cultivo e histopatología y posteriormente se efectuó lavado profuso con solución salina y hemostasia adecuada. No se indicó cierre asistido por vacío, debido a que no existía pérdida significativa de tejido ni necrosis muscular que ameritara un debridamiento extenso; además, no hubo sangrado activo ni exudado que justificara terapia de presión negativa.

En el DH 11, tras recibir el informe histopatológico y considerando la sensibilidad del *Staphylococcus* previamente aislado, se prescribió linezolid 600 mg VO cada 12 horas por 10 días (de los cuales dos días se administraron en hospitalización), asociado a celecoxib 200 mg VO cada 12 horas y curaciones diarias. Se eligió linezolid por su excelente biodisponibilidad oral, equivalente a la formulación endovenosa, lo que permitió continuar el tratamiento de forma ambulatoria. En contraste, la vancomicina requiere monitoreo estrecho de la función renal y de los niveles plasmáticos, mientras que la rifampicina exige vigilancia de la función hepática, aspectos poco factibles en un paciente procedente de una zona rural con dificultad para acudir a controles frecuentes. El perfil de sensibilidad antimicrobiana del aislamiento se presenta en el material suplementario.

### Seguimiento y resultados

El paciente fue dado de alta luego de 14 días de hospitalización. En un control a los tres meses posintervención se observó una cicatriz de aproximadamente 10 cm de longitud, con bordes adecuadamente afrontados, además de persistencia de dos úlceras en la lesión y remisión parcial de la induración (figura 3).El paciente refirió leve mejoría de dolor y persistencia de limitación funcional al caminar.

**Figura 3:** Control a los tres meses posintervención. Se observa cicatriz lineal irregular en rodilla derecha con máculas hiperpigmentadas residuales.

La evolución de los hallazgos clínicos y los estudios realizados se detalla en la figura 4, donde se muestra una línea de tiempo con los eventos más relevantes del caso.

**Figura 4:** Línea del tiempo que muestra la historia de la enfermedad del paciente desde su inicio hasta su egreso del servicio.

RMN: resonancia magnética nuclear, HSR: Hospital Santa Rosa

## DISCUSIÓN

La botriomicosis forma parte de las entidades denominadas pseudomicosis, un conjunto de infecciones crónicas que aparentan ser causadas por hongos, pero tienen origen bacteriano. Esta enfermedad suele presentarse con mayor frecuencia en pacientes inmunosuprimidos o con comorbilidades, como diabetes mellitus, hepatopatía crónica, uso prolongado de corticoides, malnutrición, enfermedades autoinmunes y síndrome de inmunodeficiencia adquirida ^3^^,^^7^.

La fisiopatología de la botriomicosis aún no se comprende completamente, sin embargo, la mayoría de casos reportados ocurren después de un traumatismo inoculante en zonas expuestas como cabeza, cuello y extremidades, lo cual desencadena un desequilibrio entre el factor de respuesta del huésped al agente extraño. Un inóculo intermedio con bacterias de baja virulencia favorece la cronificación de la lesión y la formación de los gránulos. Estos se caracterizan microscópicamente por el fenómeno de Splendore-Hoeppli, una matriz hialina periférica eosinófila (depósitos de IgG y complemento C3) que rodea a un centro basófilo (bacterias no filamentosas) ^8^.

Se han descrito dos formas de la enfermedad: cutánea y visceral. La forma cutánea suele manifestarse con lesiones nodulares solitarias o múltiples, abscesos, fístulas o úlceras. Pueden presentar secreción purulenta, gránulos blanco-amarillentos o placas induradas, las cuales pueden extenderse a músculo o hueso ^4^. La forma visceral es menos frecuente y su principal localización es pulmonar. El síntoma más común son los episodios recurrentes de hemoptisis. Por ello, suele confundirse inicialmente con tuberculosis, actinomicosis, aspergilosis o neoplasias pulmonares ^9^.

El diagnóstico de botriomicosis se establece identificando bacterias no filamentosas a partir de lesiones clínicamente compatibles. Puede realizarse mediante microscopía de los gránulos secretados, cultivo del exudado purulento o biopsia del tejido afectado ^10^. Se recomienda utilizar las tinciones de hidróxido potásico (KOH), Giemsa, Ziehl-Neelsen, entre otros; así como cultivar en agares para bacterias y hongos. De esta forma pueden descartarse otros diagnósticos diferenciales como actinomicosis, micetomas, tuberculosis y nocardiosis ^11^.

La enfermedad predomina en varones y en personas de mediana edad. La mayoría de los casos reportados corresponden a pacientes procedentes de zonas rurales, especialmente agricultores, con factores de riesgo como tabaquismo. Asimismo, varios reportes describen como antecedentes comunes la presencia de traumatismos previos y un tiempo de enfermedad mayor de 30 días ^3^^,^^11^^-^^14^.

En este caso, el paciente presentó antecedente de leishmaniasis cutánea, por lo que planteamos que las lesiones iniciales pudieron haber favorecido la infección secundaria por *Staphylococcus*, actuando como puerta de entrada para este microorganismo habitualmente presente en la piel. Tras recibir tratamiento para leishmaniasis, la infección estafilocócica habría persistido y progresado hacia una botriomicosis cutánea. Esto explicaría que, luego de completar el tratamiento antiparasitario, el frotis resultara negativo para leishmaniasis pese a la persistencia de lesiones cutáneas clínicamente sugestivas, manteniéndose inicialmente el diagnóstico diferencial erróneo de leishmaniasis activa.

Otra posibilidad es la coexistencia simultánea de ambos microorganismos, situación que se ha reportado previamente en Perú, donde se ha descrito coinfecciones por *Staphylococcus* con *Peptoestreptococcus*, *Actinobacillus* e incluso con enfermedades parasitarias como *Strongiloides*^5^. Desde el punto de vista epidemiológico, si bien se trata de un paciente masculino proveniente de una zona rural, carecía de factores de riesgo tales como diabetes u algún estado de inmunosupresión, igualmente en el interrogatorio negó el antecedente traumático precedente al inicio del cuadro reportado por la literatura.

Nuestro hallazgo coincide con un caso reportado en Lambayeque, en un agricultor con afectación del pie derecho por fístulas dorsales con secreción maloliente y gránulos blanco-amarillentos, de cinco años de evolución. En este caso se aislaron *Staphylococcus aureus* y *Actinobacillus*, con compromiso óseo metatarsiano. El paciente recibió rifampicina 600 mg y cotrimoxazol cada ocho horas durante dos meses, con mejoría parcial. Sin embargo, se rehusó al tratamiento quirúrgico y no acudió a los controles posteriores, lo que resalta la limitación del seguimiento en terapias prolongadas, dada la posibilidad de abandono ante mejoras iniciales ^4^.

En relación al tratamiento antibiótico, dependerá de la disponibilidad y el perfil de sensibilidad del agente aislado (*Staphylococcus haemolyticus)*, en el presente caso se optó por aplicar linezolid 600 mg en tabletas cada 12 horas por 10 días, sin embargo, en otro caso debido a su perfil de sensibilidad, se optó por el uso cefadroxilo oral y ácido clavulánico (500 mg, dos veces al día) durante 10 días junto con rifampicina (600 mg, una vez al día) por dos semanas ^15^. Un reporte de caso en Taiwán de una paciente de la tercera edad con mayores comorbilidades que el del presente reporte, también optó por una debridación quirúrgica de la región afectada, sin embargo, en la antibioticoterapia optó por vancomicina intravenosa por tres semanas ^16^. Ambos casos tuvieron evolución favorable. Las opciones de tratamiento antibiótico con eficacia reportada incluyen eritromicina 500 mg, dicloxacilina 100 mg, trimetoprim-sulfametoxazol 800/160 mg, cefalosporinas y clindamicina, administradas durante varias semanas y en combinación con el manejo quirúrgico ^3^^,^^6^^,^^11^^,^^17^. Entre los tratamientos invasivos, también debe considerarse el uso de cierre asistido por vacío (VAC) para el drenaje de las secreciones tras una escisión quirúrgica inicial con buenos resultados ^18^.

### Implicancias para la salud pública

Este caso demuestra que la botriomicosis puede confundirse con patologías endémicas peruanas como la leishmaniasis lo que genera retrasos diagnósticos y tratamientos inadecuados. La presentación de esta patología en un paciente inmunocompetente enfatiza la importancia de contemplarla dentro del abordaje diagnóstico diferencial de las lesiones cutáneas crónicas. Además, la identificación y el perfil de sensibilidad de *Staphylococcus haemolyticus* realza la necesidad de fortalecer la vigilancia microbiológica en el país, especialmente ante el aumento de cepas con perfiles de resistencia complejos. Debido a que gran parte de la población afectada proviene de zonas rurales con acceso limitado a servicios sanitarios se requiere estrategias que mejoren la disponibilidad de estudios microbiológicos y promuevan el uso de esquemas terapéuticos orales efectivos y accesibles.

Una limitación relevante para el reporte de este caso fue la disponibilidad incompleta y desordenada de los examenes de laboratorio e imágenes obtenidos en las atenciones previas del paciente, lo que dificultó a los autores reconstruir con precisión la secuencia diagnóstica y la progresión de la enfermedad. Asimismo, el seguimiento posterior al alta fue limitado debido a las dificultades del paciente para acudir a los controles finales por el servicio de infectología, lo que restringió la evaluación a largo plazo de la respuesta terapéutica.

En conclusión, este reporte evidencia la necesidad de considerar a la botriomicosis dentro del diagnóstico diferencial de las patologías granulomatosas crónicas no especificadas, incluso en ausencia de factores de riesgo clásicos. Asimismo, resalta la importancia de contextualizar el abordaje diagnóstico según la epidemiología de cada región. En este caso, las características clínicas de la lesión orientaron inicialmente hacia una enfermedad parasitaria endémica del Perú, como la leishmaniasis, lo que retrasó el inicio del tratamiento adecuado. Además, debido a los perfiles de resistencia cambiantes, es imprescindible contar con un antibiograma que confirme el perfil de sensibilidad del *Staphylococcus haemolyticus*, ya que la antibioticoterapia estándar que usualmente se emplea en infecciones dérmicas (betalactámicos) podría resultar insuficiente. Finalmente, aunque debe reservarse como última alternativa terapéutica, el abordaje quirúrgico no debe descartarse en casos de lesiones extensas con escasa respuesta a la antibioticoterapia.
